# Method Comparison of Erythrocyte Sedimentation Rate Automated Systems, the VES-MATIC 5 (DIESSE) and Test 1 (ALIFAX), with the Reference Method in Routine Practice

**DOI:** 10.3390/jcm13030847

**Published:** 2024-02-01

**Authors:** Michele Cennamo, Loredana Giuliano, Gloria Arrigoni, Valentina Fardone, Roberta Russo, Luca Maria De Tomasi, Fabio Bertani, Gaetano Cammarota, Giovanni Brunetti, Lucia Del Vecchio, Michelarcangelo Partenope

**Affiliations:** 1 Department of Translational Medical Sciences, University of Naples “Federico II”, 80126 Naples, Italy; 2 Clinical Pathology and Microbiology Unit, Laboratory Analysis, ASST Lariana, Hospital Sant’Anna, 22100 Como, Italy; loredana.giuliano@asst-lariana.it (L.G.); gloria.arrigoni@asst-lariana.it (G.A.); valentina.fardone@asst-lariana.it (V.F.); roberta.russo@asst-lariana.it (R.R.); luca.detomasi@asst-lariana.it (L.M.D.T.); fabio.bertani@asst-lariana.it (F.B.); michele.partenope@asst-lariana.it (M.P.); 3 Department of Diagnostic Innovation, Fondazione IRCCS Istituto Nazionale dei Tumori, 20133 Milan, Italy; gaetano.cammarota@istitutotumori.mi.it; 4 Department of Diagnostics and Public Health, Section of Clinical Biochemistry, University of Verona, 37134 Verona, Italy; giovanni.brunetti@aovr.veneto.it; 5 Department of Nephrology and Dialysis, ASST Lariana, Hospital Sant’Anna, 22100 Como, Italy; lucia.delvecchio@asst-lariana.it

**Keywords:** erythrocyte sedimentation rate (ESR), ICSH, Westergren method, VES-MATIC 5, Test 1

## Abstract

**Background**: The erythrocyte sedimentation rate (ESR) is a routine and aspecific test that is still widely used. The reference-manual method for ESR determination is the Westergren method. The VES-MATIC 5 is a novel, fully automated, and closed system based on a modified Westergren method. This study conceived the aim of comparing two ESR analytical analysers, Test 1 and the VES-MATIC 5, with the reference method in routine practice. **Methods**: This study included 264 randomly analysed samples. A comparison between the two methods and Westergren was performed, and they were evaluated for inter-run and intra-run precision. In addition, we investigated possible interferences and different sensitivities to conventional analytes. **Results**: The comparison of methods by Passing–Bablok analysis provided a good agreement for both systems, with a better correlation for VES-MATIC 5 (*p* = 0.96) than Test 1 (*p* = 0.93), and sensitivity studies did not show any significant influence. **Conclusions**: The VES-MATIC 5 analyser demonstrated excellent comparability with the reference method, and it had better performance than Test 1. It can be employed in routine practice, bringing advantages such as a reduction in the probability of human error compared to the manual method, as well as an increase in operator safety and environmental protection.

## 1. Introduction

The erythrocyte sedimentation rate (ESR) is a common haematologic test used to identify and monitor increased inflammatory activity in the body [1].

The ESR describes the dynamic biological behaviour of a suspension of red blood cells in plasma. The ESR occurs in three distinct phases when observed in a vertical tube containing anticoagulated blood for a period of one hour. The first one is the “single-cell fall”: an initial period of aggregation during which rouleaux are formed and the sediment rate is low. The second one, the “decantation phase”, is the true sedimentation phase. During this period, sedimentation occurs at a constant rate because erythrocytes fall more rapidly. In the final phase, “cell packing”, erythrocyte aggregates pile up at the bottom of the tube, and the sedimentation again slows [2,3,4]. If ESR values are plotted as a function of time, they draw a sigmoid curve. Notably, the third phase is affected by two major factors: red-cell surface charges and the presence of high molecular weight proteins, especially when positively charged. Indeed, erythrocytes typically have net negative charges and, therefore, repel each other. The presence of acute-phase proteins, which are positively charged, increases the dielectric constant in the blood and neutralises the negative charges on the surface of red blood cells, increasing the ESR [5,6,7].

The application of the ESR in clinical diagnostics was described by Biernacki, who modified his method to read the ESR using a capillary pipette called a “microsedimentator” (of his own design) instead of the original glass cylinder [8]. In this way, the scientist measured the ESR after sampling capillary blood from the tip of the finger, reading results after 60 min [9]. However, the ESR was standardised many years later by Alf Vilhelm Albertsson Westergren [8]. Westergren introduced sodium citrate as an anticoagulant for blood sampling in ESR tests and defined detailed standards for ESR tests.

The International Council for Standardization in Haematology (ICSH) later recommended Westergren’s method as the gold-standard reference because it is reliable, reproducible, and sensitive [10].

Nevertheless, there have been significant variations in the methodology used to perform ESR testing from the beginning. These variations induced ICSH to publish new recommendations improving the existing procedures several times, for example, in 1977 and 1988 [11,12]. Only in 1993, ICSH published new recommendations to ensure the standardisation and comparability of data obtained in different laboratories throughout the world and to improve the precision and accuracy of the test [13]. The ESR is not specific for a single analyte because increases are observed in very different conditions, such as autoimmune diseases, infections, or tumours [5,14,15,16,17,18]. An elevated ESR is, for example, a key diagnostic criterion for polymyalgia rheumatica and temporal arteritis, even if normal values do not preclude these conditions [19,20]. Again, a high ESR could be correlated with an overall poor prognosis for various types of cancer and metastatic disease [21,22]. Finally, extreme elevations of the ESR are often associated with infections [23,24].

Nevertheless, the ESR has long been used as a “sickness indicator” due to its reproducibility and low cost, often in combination with other tests [14].

Several new automated or semi-automated methods have become available in the last thirty years. Continuous technical innovations adopted by the new instruments have significantly improved existing procedures, delivering superior performance. The principal innovations were aimed at automation, enabling independence from the operator and guaranteeing results which are traceable to the reference method [10,25]. Moreover, the use of automated and closed systems increases the safety of operators. Some analysers allow the use of specimens collected in ethylenediamine tetraacetic acid (EDTA) and in the same tube as the CBC, optimizing the workflow and reducing the turnaround time of the test. The use of EDTA samples preserves red blood cell morphology, and it increases their stability without interfering with the mechanisms of erythrocyte sedimentation. In addition, EDTA-anticoagulated blood samples are suitable for other haematological tests on the same single specimen [26].

A recently proposed automated and closed ESR system, the VES-MATIC 5 (DIESSE, Diagnostica Senese S.p.A., Monteriggioni, SI, Italia), has been proposed to measure the real RBC sedimentation. According to the proposed classification of ESR analysers [10], the VES-MATIC 5 can be considered a modified Westergren method. It analyses samples collected in closed EDTA tubes with a completely automated process. It also uses artificial intelligence (AI) to avoid preanalytical errors, such as unfilled tubes.

On the other hand, Test 1 (Alifax s.r.l., Polverara, PD, Italy) exploits another technology, which is based on capillary photometry, allowing ESR measurement in a few minutes [10].

We conceived and designed this study with the aim of comparing our previous ESR analytical method, Test 1, and the new one, the VES-MATIC 5, with the gold standard, the Westergren method. This evaluation became necessary when we switched our Test 1 analyser for a VES-MATIC 5 analyser after the award of tender in our hospital, to ensure that the new technology can provide the same performance as the old. In addition, this is the first study comparing the three methods.

## 2. Materials and Methods

### 2.1. Sample Collection

The study included 264 randomly analysed residual blood samples from patients admitted to Hospital Sant’Anna, Como, Italy, between March and April 2023. All blood samples were leftovers from our daily withdrawals, including both ambulatory and hospitalised patients. The samples, residual and refused and all collected after routine analysis, were obtained from 135 females and 129 males (50.8% and 49.2%, respectively), with a mean age of 54.04 ± 24.54 years; median age was 59.00 years and mode was 67 years.

Samples were collected in Monovette SARSTEDT tubes (K3-EDTA, 2.6 mL) and analysed within 4 h of collection.

The study was conducted respecting the Declaration of Helsinki. It complied with the obligatory validation protocol before introducing a new analyser in routine practice, according to the International Organization for Standardization 15189:2022 (ISO 15189:2022).

### 2.2. VES-MATIC 5

VES-MATIC 5 (DIESSE, Diagnostica Senese S.p.A., Monteriggioni, SI, Italy) is an automated instrument for ESR determination in continuous loading mode. It is designed to work with the same rack as the CBC instruments. All samples, which were collected in EDTA anticoagulant tubes, were loaded directly into the analyser. After automatic mixing of the tubes under controlled temperature, samples were inserted in the analysis compartment to estimate the sedimentation rate using an optoelectronic light source. ESR was evaluated before and after real sedimentation in 20 min by optical readings and converted to Westergren data without sample manipulation and waste production. The declared manufacturer throughput is 190 samples/hour. The VES-MATIC 5 belongs to the group of instruments based on modified Westergren method [10].

Every day, before the routine analytical measurements, two levels of quality controls (normal and abnormal) composed of stabilised human blood were processed and automated archived to elaborate Levey–Jennings charts, standard deviations, CV%, and means using ESR Control Cube (DIESSE, Diagnostica Senese S.p.A. Monteriggioni, SI, Italy).

### 2.3. Test 1

Test 1 (Alifax s.r.l., Polverara, PD, Italy) is an automated instrument for ESR evaluation based on the alternative Westergren method. Using the EDTA tube and the rack intended for CBC instruments, it utilises **quantitative capillary photometry**.

The sample rate necessary for the analysis (175 uL) is transferred into a capillary tube by a fixed needle, and it flows in a transparent capillary inside the instrument and is kept at 37 °C.

The aggregation capacity of erythrocytes is measured by a photometer in 20 s and converted to Westergren data. The process generates waste, and it is necessary to clean needles with company solutions. The declared manufacturer throughput is of 150 samples/hour.

Every day, before the routine analytical measurements, we processed three levels of quality controls (low, medium and high) with a Latex Control kit (Alifax s.r.l., Polverara, PD, Italy) composed of synthetic latex solution. The control samples had known turbidity, and the values obtained were elaborated by dedicated software, ensuring accuracy and sensitivity.

### 2.4. Westergren Method

The manual Westergren method is the international reference procedure for ESR measurement. The standard was performed according to ICSH recommendations, using a diluted sample [13]. Briefly, the EDTA-anticoagulated blood was diluted, adding 1 mL of fresh blood (ratio of 1:4) to 0.250 mL of trisodium citrate dihydrate solution (3.8%). The dilution was gently mixed by complete inversion of the tube 20 times, and the sample was aspirated into a graduated circular glass pipette with a diameter of 2.55 mm. The pipette was mounted vertically on a supporting rack, allowing RBCs to pack at a constant temperature (20–25 °C) and away from external laboratory influences such as vibrations, heat, and direct sunlight. After 1 h, the sedimentation of RBCs was measured by visual inspection, quantified using the scale on the pipette, and recorded in mm/h.

All Westergren analyses were performed by a single analyst to minimise pipetting and reading variations.

### 2.5. Method Comparison Study

EDTA blood samples were analysed using the laboratory routine Test 1 analyser. After finishing the routine’s request for the specific sample, the same samples were processed by the VES-MATIC 5 instrument. Subsequently, 1 mL from each sample was manually added to sodium citrate solution in a ratio of 4:1, according to the ICSH recommendations for the Westergren method.

ESR analytical range was from 1 to 140 for VES-MATIC 5, from 2 to 120 for Test 1, and 1 to 200 for Westergren method.

Complete blood counts (CBCs) were performed on Sysmex XN-10 automated haematology analyser (Sysmex Corporation, Kobe, Japan).

### 2.6. Precision Study

Inter-run precision was evaluated using commercial quality control (QC) samples using ESR Control Cube for VES-MATIC 5 and Latex Control kit for Test 1. According to the ICSH 2017, the QC samples were analysed three times a day on five consecutive days.

Intra-run precision was determined by repeating measurements on 9 fresh blood samples from patients with different ESR analytical levels within the 4 h from collection. All samples were analysed on Test 1 and VES-MATIC 5 for five replicates.

### 2.7. Interferences and Sensitivity

Some kinds of possible interferences were evaluated in subgroups of patients.

Cholesterol (Col) interference was evaluated in 63 patients with cholesterol levels higher than the normal range (<190 mg/dL). The mean of the group was 232 mg/dL, standard deviation (SD) was 39 mg/dL, and median was 222 mg/dL; the lowest value was 190 mg/dL and the highest value was 396 mg/dL.

Triglyceride (TG) interference was gauged in 29 patients, all with pathological TG levels higher than 150 mg/dL (3.88 mmol/L); in this subgroup, the mean of TG levels was 194 mg/dL (5.02 mmol/L) with an SD of 40; the median was 180 mg/dL, the lowest value was 151 mg/dL (3.9 mmol/L), and the highest value was 307 mg/dL (7.93 mmol/L).

Anaemia interference was assessed in 78 patients with haemoglobin (Hb) levels lower than 120 g/L; mean Hb was 101 g/L, SD was 11 g/L, and median was 103 g/L; lowest and highest values were 72 g/L and 119 g/L, respectively.

In addition, we evaluated two kinds of analytical sensitivities.

We focused on sensitivity to haematocrit (Hct) below the normal range of 37–48%, or 0.370–0.480 L/L, and we evaluated it in 98 patients. The group mean of Hct was 31.4% (0.314 L/L), SD was 3.66, median was 31.9 (0.319 L/L), the lowest value was 21.6% (0.216 L/L), and the highest was 36.9% (0.369 L/L).

Sensitivity to fibrinogen was evaluated in 60 patients. We stratified samples to include all three subgroups of ESR in the analytical range and to include fibrinogen concentrations from normal to higher levels (normal range: 150–400 mg/dL). Particularly, we focused on the subgroup with fibrinogen concentrations higher than 400 mg/dL (24 patients out of 60). The overall group showed a mean of 431 ± 232 mg/dL, median of 349 mg/dL, and the lowest and highest values were 141 mg/dL and 1098 mg/dL, respectively. The subgroup with higher fibrinogen concentrations showed a mean of 637 ± 191 mg/dL, median of 595 mg/dL, and the lowest and highest values were 424 mg/dL and 1098 mg/dL, respectively.

### 2.8. Statistical Analysis

The Bland–Altman statistical test was used to compare the ESR results obtained by VES-MATIC 5, Test 1, and the reference method and to detect the bias and limits of agreement between the three methods [27,28]. Passing–Bablok regressions were applied to evaluate the proportional differences. Spearman’s rank correlation coefficients (ρ) were calculated to establish the power of the associations between the compared data [29]. Moreover, the analysis included the mean and median values, SDs, and interquartile ranges (IQRs) (25th and 75th quartiles).

Mean values and SDs were reported to evaluate the coefficients of variation (CVs) of the method’s precision.

Statistical analysis was performed using MedCalc Statistical Software version 22.009 (MedCalc Software bv, Ostend, Belgium; https://www.medcalc.org, accessed on 26 January 2024).

## 3. Results

### 3.1. Method Comparison Study

Two hundred sixty-four patients were enrolled to evaluate the performance of both devices compared to the reference method. The mean ESR values were 33.9 mm/h (95% CI: 29.7 to 38.0 mm/h) for the gold-standard Westergren method, 34.5 mm/h (95% CI: 30.3 to 38.7 mm/h) for VES-MATIC 5, and 38.9 mm/h (95% CI: 34.8 to 43.0 mm/h) for Test 1.

The median ESR value was 18 mm/h (IQR: 7–50 mm/h) using the Westergren method, 19 mm/h (IQR: 6–59.2 mm/h) with VES-MATIC 5, and 26 mm/h (IQR: 10.7–60 mm/h) with Test 1.

We compared the two methods (VES-MATIC 5 and Test 1) versus the reference one, the Westergren technique.

**Figure 1:**
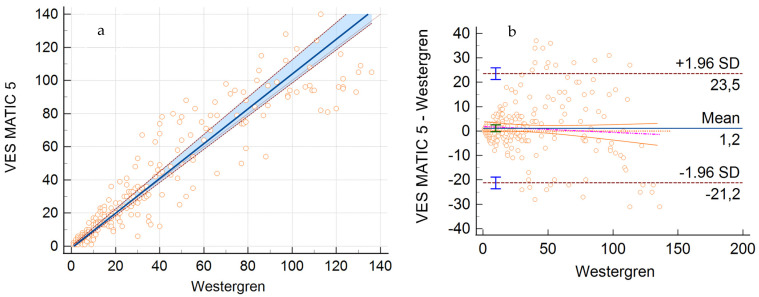
Correlation analysis between VES-MATIC 5 and Westergren method. (**a**) Regression equation calculated using Passing–Bablok’s correlation. (**b**) Bland–Altman plot: mean bias, upper and lower limits.

The comparison between VES-MATIC 5 and Westergren method by Passing–Bablok analysis showed a suitable agreement without systematic or proportional differences, with some little differences on the part of VES-MATIC 5. The regression equation was y = −0.8 (95% CI: −1.6 to 0.0) + 1.1 (95% CI: 1.0 to 1.2) x. The Spearman rank correlation coefficient (*p*) was 0.96, with a 95% CI from 0.95 to 0.97 (Figure 1a).

A mean bias of 1.2 (95% CI: −0.2 to 2.6) was obtained by Bland–Altman analysis; a value of 23.5 for the upper limit and −21.2 for the lower limit can be considered acceptable, implicating a non significant bias based on clinical criteria (Figure 1b).

**Figure 2:**
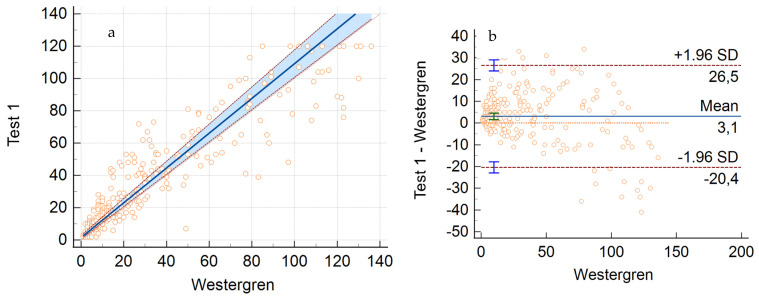
Correlation analysis between Test 1 and Westergren method. (**a**) Regression equation calculated using Passing–Bablok’s correlation. (**b**) Bland–Altman plot: mean bias, upper and lower limits.

On the other hand, the comparison between Test 1 and the Westergren method showed the following regression equation: y = 1.6 (95% CI: 0.8 to 2.9) + 1.1 (95% CI: 1.0 to 1.1) x (Figure 2a). The Spearman rank correlation coefficient (*p*) was 0.93, with a 95% CI that was 0.91 to 0.94.

A mean bias of 3.1 (95% CI: 1.6 to 4.6) was obtained by Bland–Altman analysis; the values of 26.5 for the upper limit and −20.4 for the lower limit can be considered acceptable, implicating a nonsignificant bias based on clinical criteria (Figure 2b).

### 3.2. Precision Studies

In Table 1, the results from the intra-run precision study are presented. There were nine samples evaluated, with five replicates. ESR results are reported as mean value, SD, CV%, and the minimum and maximum values obtained.

For both methods, we did not report very low ESR sample values but those still in normal range. This choice was made due to the intrinsic bias of the mathematical calculations of SD and CV% for very low values. In fact, in very low values, even for millesimal differences, for example, only 2–3 mm/h, SD and CV% (calculated as mean/standard deviation) increase exponentially.

Regarding VES-MATIC 5, the five replicate measurements of the specimens showed an excellent level of precision of below 15% (CV < 15%), except for among the specimens with very low results. On the other hand, regarding Test 1, the results showed a good level of precision below 15% (CV < 15%), except for one specimen.

Table 2 shows the inter-run precision. For the VES-MATIC 5, the evaluation was determined by analysing the QC material three times on five consecutive days, both in the normal and abnormal ranges. The inter-run precision was around 30% (CV 32%) for the normal levels, even with very low values, and 3% (CV 3.3%) for abnormal levels. As confirmed with Grubbs’ test, there were no outliers.

As for the samples, for a low level of QC control, the CV% was high due to millesimal differences from low ESR values, as can be seen by the means and the SDs; therefore, the inaccuracy was minimal, and the systemic bias was very low.

The QC material employed for Test 1 was analysed simultaneously for low, middle, and high ranges. As expected, due to the synthetic matrix of the latex solution, the results were very precise and, consequentially, CVs were <1%, 3.3%, and 2% for low, middle, and high range, respectively. Also, in this case, the Grubbs test did not reveal any outliers.

The difference in CVs can be explained by the different types of samples used as controls. Indeed, Test 1 uses latex controls, while control samples on the VES-MATIC 5 use stabilised human blood.

### 3.3. Interferences and Sensitivity

The interferences we evaluated returned results of interest for clinical practice.

The assessment of interferences that lipemic samples may have was conducted by evaluating the ESR results with the different methods among patients with known values of Col and TGs.

In the comparison of Col samples (Table 3), the PB regression equation was y = −1.0 (95% CI: −2.2 to 1.2) + 1.0 (95% CI: 0.9 to 1.2) x for the comparison between VES-MATIC 5 and the Westergren method. The Spearman rank correlation coefficient (*p*) was 0.92, with a 95% CI of 0.88 to 0.95.

On the other hand, for comparison between Test 1 and the Westergren, indeed, the PB equation was y = 0.0 (95% CI: −2.8 to 4.2) + 1.4 (95% CI: 1.0 to 1.8) x with a Spearman rank correlation coefficient (*p*) of 0.86, with a 95% CI of 0.78 to 0.91.

Regarding the evaluation of TG interference, the PB analysis showed the equations y = −1.2 (95% CI: −4.6 to 2.5) + 1.06 (95% CI: 0.9 to 1.3) x and y = 5.0 (95% CI: −0.9 to 9.1) + 1.0 (95% CI: 0.7 to 1.4) x and Spearman rank correlation coefficients (*p*) of 0.96 and 0.84, for VES-MATIC 5 and Test 1 versus Westergren, respectively (Table 4).

Another possible interfering factor is anaemia.

In this case, in the equation obtained in PB analysis, the correlation between VES-MATIC 5 and the Westergren was y = 1.5 (95% CI: −1.1 to 5.8) + 0.9 (95% CI: 0.8 to 1.0) x, and the Spearman coefficient (*p*) was 0.93 (95% CI: 0.89 to 0.95).

Conversely, the comparison between Test 1 and the Westergren method showed regression correlation with the following equation: y = 1.1 (95% CI: −2.2 to 5.7) + 1.0 (95% CI: 0.9 to 1.1) x. The Spearman rank correlation coefficient (*p*) was 0.91, with a 95% CI of 0.86 to 0.94 (Table 5).

The diagnostic sensitivity studies aimed to understand the behaviour of the methods in routine-specific samples; in particular, we focused on low Hct levels and high levels of Fib.

Focusing on samples with low Hct levels, we found an excellent correlation between the three methods. As the result for the correlation between VES-MATIC 5 and the Westergren, we received the following equation: y = 0.0 (95% CI: −1.3 to 0.0) + 1.0 (95% CI: 0.9 to 1.1) x, with a Spearman *p* of 0.94. As the result for the correlation between Test 1 and the Westergren, we received the equation y = 4.2 (95% CI: −0.6 to 8.5) + 0.95 (95% CI: 0.9 to 1.0) x, with a Spearman *p* of 0.92 (Table 6).

The sensitivity to increasing amounts of fibrinogen was determined. We found similar sensitivities with the VES-MATIC 5, Test 1, and the Westergren reference method.

In the total group of 60 patients, the assessment was evaluated using the Passing–Bablok statistical method. The obtained equations were y = 0.4 (95% CI: −2.6 to 2.2) + 1.1 (95% CI: 1.0 to 1.2) x and y = 2.0 (95% CI: −0.9 to 4.3) + 1.0 (95% CI: 0.9 to 1.2) x, with Spearman rank correlation coefficients (ρ) of 0.93 (95% CI: 0.89 to 0.96) and of 0.85 (95% CI: 0.76 to 0.91) for VES-MATIC 5 and Test 1, respectively (Table 7). The subgroup with high fibrinogen levels was made up of only 24 patients. Consequently, it was difficult to assess a Bland–Altman correlation. For this reason, we merely evaluated the statistical value. Nevertheless, in our opinion, even in the case of high fibrinogen values, there is no interference with the method under consideration (Table 7).

## 4. Discussion

The ESR is a nonspecific sickness index, which is not diagnostic of any particular disease. However, an elevated ESR may indicate the presence of inflammation, infection, rheumatologic disease, or cancer. For this reason, this simple test, along with other inflammatory markers, such as C-reactive protein and the patient’s clinical history, can support the clinician in making a more accurate diagnosis.

The ESR is still of clinical value in diagnosing and monitoring response to therapy in autoimmune diseases, such as rheumatoid arthritis and systemic lupus erythematous [17,30].

The traditional Westergren method has been replaced in most laboratories with novel automated instrumentations for many reasons: reduction in the exposure of laboratory personnel to infectious diseases, the ability to use the EDTA tubes destined to CBCs without an additional sampling, and the faster turnaround time. In addition, increased automation reduces the probability of human error, increases economic efficiency, and allows instruments to be interfaced with laboratory middleware [10,31].

In this study, we evaluated the analytical performance of the standard hospital routines of two automated ESR analysers, VES-MATIC 5 and Test 1, comparing them with the Westergren reference method.

Both use undiluted EDTA samples. This allows the use of the same specimen collected for CBC. Moreover, these analysers utilise the Sysmex racks (identified with univocal numbers and barcodes) where blood tubes are put after being accepted by the laboratory. This hallmark prevents the sample’s original position from being changed and makes it easy to find a specific specimen.

Both analysers have a throughput of no fewer than 150 samples/hour and load samples on board differently: VES-MATIC 5 has eighteen workstations on which to place Sysmex racks; instead, Test 1 has only four. In addition, Test 1 has no internal data storage, whereas VES-MATIC 5 has it; this allows the technician not to print any result and not to waste thermal paper.

According to the ICSH 2017 classification [10], the VES-MATIC 5 is an analyser based on the modified Westergren method, while Test 1 is considered an alternative method. Using an optical reading system, VES-MATIC 5 does not pipette the sample to analyse it, so there is no waste production. However, Test 1 needs to take a small amount of blood from the primary tube. After heating, the analyser proceeds to make the ESR measurement by quantitative capillary photometry. Working on closed blood tubes, VES-MATIC 5 has the advantage of not requiring routine cleaning and maintenance washes of the instrument.

To compare the accuracy of the two instruments and the reference method, ESRs were measured in 264 patients randomly chosen from routine hospital practice.

The ESR means obtained were 33.9 mm/h for the gold-standard Westergren method, 34.5 mm/h for VES-MATIC 5, and 38.9 mm/h for Test 1, highlighting an over-estimation of Test 1 results with respect to the other methods. In all the comparative analyses carried out, VES-MATIC 5 showed an excellent correlation with the Westergren method that was better than Test 1. Our results showed a correlation coefficient (ρ) of 0.96 (CI 95%: 0.95 to 0.97) between VES-MATIC 5 and the Westergren and a correlation coefficient (ρ) of 0.93 between Test 1 and the Westergren, with a 95% CI of 0.91 to 0.94. This result perfectly aligns with previous studies [32,33,34] and confirms the analytical validity of both instruments.

The precision was also evaluated to ensure the repeatability and reproducibility of both analysers.

The intra-run precision was assessed using nine fresh blood samples from patients with different ESR analytical levels, which were analysed for five replicates, and inter-run precision, using commercial quality control (QC) samples was analysed three times a day on five consecutive days.

Both VES-MATIC 5 and Test 1 showed excellent levels of precision that were less than 15% (CV < 15%) for ESR values > 20 mm/h, while with very low ESR results, we obtained higher values. These CVs resulted from the arithmetic issue caused by small ESR values, not the instruments’ unsatisfactory performance. Moreover, as reported in Table 2, none of the samples with low ESRs moved the normal analytical range to a pathological ESR despite their high CVs, as confirmed by the minimum and maximum values.

Regarding the inter-run precision, we only observed differences in CVs for low ESR levels. They could be ascribed to the different types of control samples (synthetic latex for Test 1 and stabilised human blood for the VES-MATIC 5) [35,36,37]. In particular, the Test 1 results were very precise, with CVs < 1%, 3.3%, and 2% for low, middle, and high ranges, respectively. VES-MATIC 5 had a CV of 32% for normal levels due to very low values and 3% for abnormal levels. These data are perfectly acceptable, as has already been described in the literature [37,38,39,40,41].

ESR can be influenced by lifestyle (physical activity, smoking, and alcohol consumption) or by metabolic disorders [42,43]. In addition, preanalytical factors can generate a diagnostic error, too [44,45]. Specifically, lipemic and haemolysed sera are often found in the everyday practice of clinical laboratories; these abnormalities can cause significant interferences in the analytical results. The obtained findings, in association with other sensitivities, for example, fibrinogen sensitivity, could drive the clinician to an incorrect interpretation of the results [31,42,44,46,47,48,49]. For this reason, we tried to investigate the main interferences and the sensitivities that more frequently occur in hospital routines when ESRs are determined.

First, we investigated lipid interference, measuring samples from 63 patients with high Col levels and 29 patients with high TG levels.

The correlation coefficients (ρ) between VES-MATIC 5 and Westergren were 0.95 and 0.96 for Col and TGs, respectively, maintaining the good correlation calculated for total samples. Test 1 showed a decrease in its performance in the presence of these interferences (ρ = 0.86 for Col and ρ = 0.84 for TGs). Compared to other studies, no significant differences in ESR values were observed in Col and TG interference evaluations with the two methods. However, this secondary analysis is limited by the small number of samples assessed [50,51,52].

Anaemia can affect the erythrocyte’s sedimentation, causing an acceleration of the global phenomenon. We evaluated the effect of anaemia by employing samples from 78 patients with Hb levels ranging from 72 to 119 g/L. In this study, VES-MATIC 5 and Test 1 compared well against the Westergren method through statistical regression (*p* = 0.93 and *p* = 0.91), with no significant systematic difference, which is in agreement with previous studies [33,43,53].

In addition, we evaluated sensitivity to a low range of Hct in 98 patients to investigate the sensitivity of anaemia further. We found a very good correlation between the three methods; particularly, the Spearman’s *p* was 0.94 between VES-MATIC 5 and the Westergren method and had a *p* of 0.92 for the Test 1 comparison. Also, for this analysis, our data aligned with the literature [47,54], highlighting that both analysers are independent of anaemic status.

Fibrinogen is one of the main proteins responsible for aggregation and is among the most critical variables affecting ESR values, along with haematocrit and erythrocyte variations [14,15,16,17,55,56,57,58,59]. Its effects are most evident in the second phase of the erythrocyte sedimentation process, where fibrinogen, due to its positive charge, mediates the formation of large aggregates and enhances the settling of RBCs [59].

Sensitivity to fibrinogen was studied in 60 patients with different diseases who were stratified to include three subgroups according to their fibrinogen concentrations. The mean concentration in the group with high fibrinogen levels was 636.58 mg/dL. The VES-MATIC 5 response to each level of fibrinogen concentration was in agreement with the Westergren, indicating that its technology is interchangeable with the gold-standard method (*p* = 0.93).

## 5. Conclusions

The ESR is not a measure of a single analyte but rather a result of a complex biophysical phenomenon with a clinical utility. ESR results can be affected in some particular cases by different methodological principles [15,40,41,42]. However, the implementation of automated ESR technologies undoubtedly provides faster and more reproducible determination results, optimizing time and resources and often lowering the level of biological risk for operators.

In conclusion, this study assessed the analytical validity of Test 1 and VES-MATIC 5 analysers. Moreover, our findings attest to the excellent comparability with the gold-standard method and better performance of VES-MATIC 5 than Test 1. We believe that the VES-MATIC 5 can be employed appropriately for routine purposes, bringing the mentioned advantages.

## Figures and Tables

**Table 1:** Intra-run precision results.

	Test 1					VES-MATIC 5				
Sample (Rep n = 5)	Average	Stdev	cv%	MIN	MAX	Average	Stdev	cv%	MIN	MAX
1	23	2.2	9	21	26	22	2.5	11	18	24
2	21	5.7	27	14	27	21	2.5	12	19	25
3	33	1.9	6	30	35	25	2.5	10	23	29
4	39	2.3	6	36	42	30	0.5	2	29	30
5	60	5.2	9	53	67	76	2.5	3	74	79
6	61	2.4	4	58	63	75	6.5	9	66	84
7	53	5.6	11	48	62	67	8.4	13	57	77
8	72	4.0	6	66	77	80	5.2	7	72	86
9	106	5.6	5	100	113	88	13.0	15	65	96

**Table 2:** Inter-run precision results.

	VES-MATIC 5		Test 1		
	Normal	Abnormal	Low	Mid	High
Mean ± SD	3 ± 1.0	51 ± 3.8	8 ± 0.0	18 ± 0.6	61 ± 1.2
CV%	32%	7%	0%	3.3%	2.0%

**Table 3:** Results of method comparison for samples with high cholesterol levels.

	Westergren	VES-MATIC 5	Test 1
Lowest value	2.0	1.0	2.0
Highest value	123.0	105.0	85.0
Arithmetic mean	21.7	22.0	26.4
Median	14.0	17.0	21.5
Standard deviation	24.6	23.5	20.1

**Table 4:** Results of method comparison for samples with high triglyceride levels.

	Westergren	VES-MATIC 5	Test 1
Lowest value	3.0	2.0	2.0
Highest value	135.0	128.0	120.0
Arithmetic mean	38.1	39.8	37.9
Median	22.0	24.0	26.0
Standard deviation	38.3	39.7	32.3

**Table 5:** Results of the method comparison for samples with Hb < 120 g/L.

	Westergren	VES-MATIC 5	Test 1
Lowest value	1.0	1.0	2.0
Highest value	136.0	124.0	120.0
Arithmetic mean	65.1	62.3	64.5
Median	67.5	63.0	63.0
Standard deviation	40.5	36.9	38.3

**Table 6:** Results of method comparison for samples with low Hct levels.

	Westergren	VES-MATIC 5	Test 1
Lowest value	1.0	2.0	2.0
Highest value	131.0	140.0	120.0
Arithmetic mean	63.1	60.6	65.0
Median	62.0	62.0	63.0
Standard deviation	37.2	35.2	34.8

**Table 7:** Results of method comparison of 24 samples with abnormal fibrinogen values.

	Westergren	VES-MATIC 5	Test 1
Lowest value	1.0	1.0	2.0
Highest value	131.0	131.0	120.0
Arithmetic mean	39.8	43.8	45.5
Median	24.5	28.0	34.0
Standard deviation	38.1	37.9	38.5

## Data Availability

We state that the data are available to the scientific community.
